# Stress Management Interventions to Facilitate Psychological and Physiological Adaptation and Optimal Health Outcomes in Cancer Patients and Survivors

**DOI:** 10.1146/annurev-psych-030122-124119

**Published:** 2022-08-12

**Authors:** Michael H. Antoni, Patricia I. Moreno, Frank J. Penedo

**Affiliations:** 1 Department of Psychology, University of Miami, Coral Gables, Florida, USA; 2 Cancer Control Research Program, Sylvester Comprehensive Cancer Center, University of Miami, Miami, Florida, USA; 3 Department of Public Health Sciences, University of Miami School of Medicine, Miami, Florida, USA; 4 Department of Medicine, University of Miami School of Medicine, Miami, Florida, USA

**Keywords:** cancer-related stressors, psychological adaptation, physiological adaptation, cognitive behavioral therapy, stress management intervention, cancer health outcomes

## Abstract

Cancer diagnosis and treatment constitute profoundly stressful experiences involving unique and common challenges that generate uncertainty, fear, and emotional distress. Individuals with cancer must cope with multiple stressors, from the point of diagnosis through surgical and adjuvant treatments and into survivorship, that require substantial psychological and physiological adaptation. This can take a toll on quality of life and well-being and may also promote cellular and molecular changes that can exacerbate physical symptoms and facilitate tumor growth and metastasis, thereby contributing to negative long-term health outcomes. Since modifying responses to stressors might improve psychological and physiological adaptation, quality of life, and clinical health outcomes, several randomized controlled trials have tested interventions that aim to facilitate stress management. We review evidence for the effects of stress management interventions on psychological and physiological adaptation and health outcomes in cancer patients and survivors and summarize emerging research in the field to address unanswered questions.

## STRESSORS AND CHALLENGES AMONG CANCER PATIENTS AND SURVIVORS

Receiving a cancer diagnosis is a profoundly stressful experience involving unique and common challenges that often introduces uncertainty, fear, and emotional distress (Singer 2018, Stanton 2006). Patients are tasked with understanding new information and making important and often complex treatment decisions. These treatment decisions occur while experiencing significant disruptions to patients’ daily lives and the social/occupational roles that often define their identity (Henoch & Danielson 2009). A diagnosis of cancer also frequently brings salient awareness of one’s own mortality and vulnerability and a degree of uncertainty that can further exacerbate negative emotional reactions (Lee & Loiselle 2012). Most cancers require intervention; however, the specific therapy or therapies and duration of treatment can vary greatly across cancer types and stages (Am. Cancer Soc. 2020a). Common targeted cancer treatments that remove or destroy malignant tissue include surgery to excise a tumor and radiation to ablate DNA in cancer cells. Common systemic therapies (i.e., treatments that target cancer cells throughout the entire body) include chemotherapy to kill rapidly growing and dividing cancer cells, hormone therapy to modify hormones like estrogen or androgens that fuel growth in specific types of cancer cells, and immunotherapy to enable a person’s own immune system to identify and attack cancer cells. Collectively, these treatments can create short- and long-term side effects and toxicities that persist well beyond treatment.

Cancer treatments can be classified into different categories based on the goal or intention of the treatment.^1^ Primary treatment refers to the first or main treatment used to eliminate or reduce traceable cancer. Adjuvant therapy is additional treatment given after primary treatment to eliminate any remaining cancer cells using either systemic or nonsystemic therapies. Neoadjuvant therapy is treatment that occurs prior to initial treatment (most typically surgery) to facilitate the primary treatment or make it more effective. Palliative treatment or treatment with palliative intent is the use of any therapy with the goal of improving quality of life (QoL; i.e., social, functional, emotional, physical functioning) and reducing the physical burden of cancer by relieving treatment side effects or symptoms related to the cancer itself. Palliative treatment can be applied to patients with any stage of cancer experiencing symptom burden; however, it is often the focal treatment for those with metastatic cancer that has spread to other parts of the body from where it originated.

An individual’s response to treatment with respect to both its effectiveness and its side effects is highly variable. Symptomatic side effects or toxicities, such as pain, nausea, fatigue, and neuropathy, can significantly reduce the tolerability of cancer treatments (a patient’s capacity to adhere to therapy) and therefore have a negative impact on QoL and psychosocial adjustment (Basch et al. 2009, Pearman et al. 2018). Cancer treatment and related disability also have a negative impact on an individual’s finances as a result of the direct and indirect costs of treatment. This economic challenge, known as financial toxicity, is highly prevalent among individuals with cancer (Carrera et al. 2018, Lentz et al. 2019) and further compromises QoL. Patients who are more recently diagnosed and those who receive adjuvant therapies are more likely to experience financial toxicity (Chino et al. 2017). Similar to other toxicities, financial toxicity reduces the tolerability of cancer treatments and has a deleterious impact on QoL and psychosocial adaptation (Carrera et al. 2018, Lentz et al. 2019). It can also result in material consequences like reduced income, increased debt, depletion of savings, and bankruptcy (Lentz et al. 2019).

Advances in cancer prevention, screening, and treatment have led to a significant increase in the number of individuals who live beyond a cancer diagnosis and the completion of curative-intent treatment (Miller et al. 2019). Although many individuals recover from the decrements in QoL that they experience during cancer treatment after treatment completion, these effects may persist long-term in some individuals or emerge for the first time months or even years later (known as late effects; Am. Soc. Clin. Oncol. 2019). The most common symptoms individuals experience are pain, fatigue, and impairments in physical functioning; however, sexual and urinary/bowel dysfunction, cognitive impairment, and sleep disturbance are also frequently reported (Kent et al. 2015, Stanton et al. 2015, Stein et al. 2008). Receiving cancer treatment can also increase the risk for subsequent cancers (known as second primary cancers), and toxicities can adversely impact the cardiovascular and reproductive systems (Demoor-Goldschmidt & de Vathaire 2019, Ganz 2001). As a result, individuals previously treated for cancer often undergo long-term surveillance by both specialists and primary care providers in order to mitigate these risks (Wilbur 2015).

In contrast to treatment with curative intent, treatment for incurable advanced or metastatic cancer is often not circumscribed to a definitive period and does not include a post–treatment completion phase as with curative-intent treatment (Am. Cancer Soc. 2020b). Furthermore, treatment plans and their intent (e.g., palliation versus life-prolonging) must be tailored to an individual’s treatment response. Due to the nature of the advanced disease, these patients experience a high degree of uncertainty and anxiety tied to multiple repeated diagnostic procedures as care teams must continuously evaluate how their cancer is responding to treatment and whether their treatment plan needs to be changed (Bauml et al. 2016, Dunn et al. 2017). Accordingly, patients may experience significant treatment-related burnout and cumulative effects of cancer treatment on symptomatic side effects and toxicities, which underlines the importance of patient-centered care and ongoing discussion with care providers regarding the goals of care and the possible benefits and side effects of cancer treatments (Langbaum & Smith 2019).

### Psychological Adaptation: Cancer-Related Distress

Given the stressors and challenges associated with diagnosis and treatment, it is not surprising that cancer can take a significant toll on emotional well-being and require sustained psychological adaptation. Anxiety and depressed mood are two of the most common emotional reactions among individuals who are undergoing cancer treatment (Jacobsen & Andrykowski 2015) or have completed treatment (Stanton et al. 2015). About 30–40% of individuals with cancer meet diagnostic criteria for anxiety and other mood disorders (Mitchell et al. 2011); however, subclinical elevations in symptoms and other forms of cancer-related distress still negatively impact QoL and should be addressed. As expected, disease severity, premorbid psychological functioning, access to care, and functional limitations typically exacerbate negative emotional reactions and compromise psychosocial adjustment following the diagnosis and treatment of cancer. Further, fear of cancer progression or recurrence is one of the most frequent and persistent concerns individuals experience following a cancer diagnosis (Koch et al. 2013, Simard et al. 2013). Anxiety in anticipation of cancer-related surveillance scans (referred to as scanxiety) is also common during and following treatment (Bui et al. 2021, Custers et al. 2021). Fear of cancer progression or recurrence and scanxiety are distressing and are associated with significantly worse QoL (Bui et al. 2021, Koch et al. 2013, Simard et al. 2013). Furthermore, uncertainty about the future and concern for close others are two supportive care needs that individuals often report have not been adequately addressed by their care team (Armes et al. 2009, Harrison et al. 2009).

### Psychological Adaptation: Resilience

Despite the considerable impact of cancer on psychological adaptation, individuals often demonstrate resilience. Facing cancer can lead to opportunities for positive change as individuals engage in efforts to find meaning in their experience (Algoe & Stanton 2009, Park et al. 2008, Stanton et al. 2006). Positive changes, such as enhanced life appreciation, improved social relationships, and a deepened sense of self and meaning that individuals attribute to stressful life experiences like cancer, have been referred to as benefit finding, post-traumatic growth, and personal growth (Helgeson et al. 2006, Tedeschi & Calhoun 2004). Most individuals who have completed cancer treatment report experiencing some level of post-traumatic growth in response to their cancer diagnosis and treatment (Jim & Jacobsen 2008, Stanton et al. 2006), which is generally associated with better psychological adaptation, including lower anxiety and depressive symptoms as well as better QoL and increases in optimism, hope, and positive affect (Algoe & Stanton 2009, Casellas-Grau et al. 2017, Rajandram et al. 2011, Stanton et al. 2006). Individuals with advanced or metastatic cancer also cite finding meaning at the end of life as important and perceive positive consequences as a result of their experience (Moreno & Stanton 2013). Importantly, the commonly used term “post-traumatic growth” is paradoxical in this context, given that advanced cancer often has an uneven course, which is not circumscribed to a definitive period with a beginning and an end as is more likely for illnesses treated with curative intent. Personal growth in the context of advanced cancer is positively associated with both distress, including depressive symptoms and cancer-specific intrusive thoughts and avoidance, and positive well-being, including optimism, positive affect, and acceptance (Moreno & Stanton 2013). This co-occurrence of personal growth with both cancer-related distress and positive well-being suggests that personal growth in this unique context is characterized by perceived positive consequences in the face of considerable demands, which may be reflected by greater negative and positive markers of psychological adaptation.

### Physiological Adaptation

The multiple stressors that cancer patients must manage, from the point of diagnosis and treatment decisions, through surgical and adjuvant treatments, and into survivorship, require a substantial amount of emotional processing and adaptation, both psychologically and physiologically. The physiological basis of adapting to stressors was initially articulated by Hans Seyle in his notion of general adaptation syndrome (Selye 1956). We know that aspects of stress responding in the face of acute short-term stressors are quite different from responses to chronic or repeated stressors, and these differences can be measured with neuroendocrine and immunological indicators that may be particularly relevant in cancer patients (Antoni & Dhabhar 2019). Generally, chronic and repeated stressors are associated with immunologic changes that promote negative health outcomes in most populations, including cancer patients (Antoni & Dhabhar 2019).

One physiological system often implicated in responses to stressors is the sympathetic nervous system (SNS), which mediates neural signaling directly from brain regions such as the locus coereleus (LC) to peripheral tissue via the sympathetic chain of neural fibers. Sympathetic neurons release norepinephrine (NE) at junctures with many vital organs, including the heart, as well as with immune-related lymphoid organs such as the lymph nodes, bone marrow, and gut-associated lymphoid tissue (GALT), which produce or house different types of immune cells that bear beta-adrenergic receptors (β-ARs) on their cell membranes (Cole et al. 2015). We now know that tumors also have nerves and β-ARs (Huang et al. 2014). This suggests that stress-related activation of the SNS with production of NE can communicate with immune cells as well as cancer cells. The SNS also controls the outflow of epinephrine (E) from the adrenal medulla into the circulation (sympathoadrenomedullary system, or SAM), and E is also capable of signaling immune and tumor cells via β-ARs. These responses may occur when stressors are acute and require an immediate, often physical, response, and hence they have been referred to as part of the fight-or-flight pattern. The SNS-mediated responses, however, are also evidenced during emotionally laden stressors and repeated stressors, and they co-occur with distress states such as depression and anxiety (Antoni & Dhabhar 2019).

Another physiological stress response system, the conservation-withdrawal response, is often associated with persisting, repeated, uncontrollable, and unpredictable stressors as well as distress states such as depression and threat/anxiety and is characterized by activation of and dysregulation of the hypothalamic-pituitary-adrenal (HPA) axis. Following the perception of uncertainty from cortical regions of the central nervous system, the hypothalamus produces corticotrophin releasing hormone (CRH), which in turn signals the pituitary to release adrenocorticotrophic hormone (ACTH) into the circulation, which signals the adrenal cortex to produce the glucocorticoid cortisol (McEwen 2002). This system may be particularly relevant to the chronic, repeated, and uncertain nature of the periods surrounding cancer diagnosis, treatment, post-treatment survivorship, and disease recurrence. Importantly, cortisol is capable of interacting with immune cells via intracytoplasmic glucocorticoid receptors to affect cellular immune programming as well as function and inflammation (Antoni et al. 2006b, Chang et al. 2022, McEwen 2002). Finally, chronic elevations in cortisol have been proposed to downregulate glucocorticoid receptors (GRs) in immune cells, effectively dampening anti-inflammatory control after immune responses and resulting in increased systemic inflammation (Miller et al. 2008).

### Psychological Stress and Neuroendocrine Regulation in Cancer

Many reviews focus on the influence of neuroendocrines on the immune system as a plausible explanation for stress effects on negative health outcomes in cancer patients (Antoni & Dhabhar 2019). These include stress effects on changes in immune cell adhesion and trafficking, cellmediated immunity, humoral immunity, lymphocyte proliferation, macrophage responses, and natural killer cell cytotoxicity, many of which have potential roles in immune surveillance of cancer cells (Antoni & Dhabhar 2019).Stress factors can also enhance inflammatory signaling on the one hand and upregulate immunosuppressive signaling on the other, which could conspire to impair the host’s ability to detect and destroy neoplastic cells (Cole et al. 2015, Falcinelli et al. 2021).

Other reviews of the field refer to a neurobiology of cancer articulating the links among psychosocial factors, the nervous system, and tumor tissue (Mravec et al. 2020). The production of NE, E, or cortisol has been proposed to mediate, in part, the effects of major chronic stressors and distress states, such as those experienced by cancer patients, on processes that promote cancer progression and metastasis, including increased cell growth/proliferation rates; enhanced blood supply to cancer cells (angiogenesis); invasion into the vasculature; increased ability of tumor cells to survive in the circulation through epithelial-to-mesenchymal transition (EMT); and protection against programmed cell suicide (apoptosis) when tumor cells detach themselves from the extracellular matrix and move into the circulation (also known as anoikis) (Chang et al. 2022). We refer the interested reader to recent comprehensive reviews of this work (Chang et al. 2022, Eckerling et al. 2021, Falcinelli et al. 2021, Mravec et al. 2020).

The nervous system is believed to influence cancer initiation and progression via DNA mutations and oncogene signaling (Falcinelli et al. 2021). The nervous system is also believed to contribute to tumor growth and metastasis via direct interactions with nerves in tumors, neurohormonal modulation of immune cell presence via SNS-mediated efflux of myeloid cells from the bone marrow (Powell et al. 2013), and up-regulated tumor cell activity (and immune-tumor cross-talk) in the tumor microenvironment. These processes can facilitate angiogenesis and tissue remodeling (via matrix metalloproteases such as MMP-9), allowing vascular invasion and spread into the circulation (Chang et al. 2022, Cole et al. 2015).

Recent work suggests that stress-related neuroendocrines may even contribute to reactivating dormant cancer cells. For instance, stress hormones activating β-ARs can stimulate release of s100A8/A9 ligands from neutrophils, which promotes activation of the receptor for advanced glycation end (RAGE) products, causing release of oxidized lipids that activate dormant cancer cells via a fibroblast growth factor pathway (Perego et al. 2020). Interestingly, in postsurgical breast cancer patients, greater serum cortisol levels are associated with greater cancer-specific distress on the one hand and greater levels of s100A8/A9 on the other (Taub et al. 2022). Some work also shows that glucocorticoids may facilitate resistance of tumor cells to cytotoxic chemotherapy, rendering some cancer treatments less effective (Chang et al. 2022). These bodies of evidence join with other models emerging in the past 20 years proposing how stressors and psychosocial factors relate to cancer incidence, progression, and metastasis across different cancers (Falcinelli et al. 2021, Lutgendorf et al. 2010).

Preclinical animal models have shown that a variety of experimental stressors (forced swimming, isolation, surgery) (Antoni et al. 2006b, Eckerling et al. 2021) can increase the likelihood of cancer progression. Using an ovarian cancer model, investigators showed that isolation stress and/or administration of the β-AR agonist isoproterenol can upregulate processes supporting accelerated tumor growth, such as angiogenesis and vascular endothelial growth factor (VEGF) production, as well as changes supporting the metastatic spread of established tumors by increased tissue invasion, anoikis, and increased tumor EMT (Chang et al. 2022, Lutgendorf et al. 2010). The activation of the SNS and release of NE have also been proposed to mediate many of these effects in models of breast cancer (Sloan et al. 2010). Parallel clinical work has related psychosocial factors such as distress, depression, and low social support with greater levels of cortisol in breast cancer patients (Chang et al. 2022) and a flatter cortisol diurnal secretion pattern in patients with renal cell carcinoma (RCC) (Cohen et al. 2012). Flatter salivary cortisol diurnal pattern has been associated with shorter survival in breast (Sephton et al. 2000), lung (Sephton et al. 2013), and renal cell (Cohen et al. 2012) carcinomas.

### Psychological Stress and Neuroendocrine-Mediated Changes in Immune Activation and Regulation in Cancer

Much work in the past 30 years has related stress processes to changes in immune system activity and regulation in cancer patients (Antoni & Dhabhar 2019). Much of the earlier work focused on associations of negative affect and depressive symptoms with in-vitro cellular immune function indicators such aslymphocyte proliferative responses (LPR), T-lymphocytehelpertype 1 (Th1) cytokine [interleukin-2 (IL-2) and interferon-gamma (IFN-γ)] production, and natural killer cell cytotoxicity (NKCC) in breast cancer patients (Andersen et al. 1998, Levy et al. 1987). More recently attention has turned to relating stress factors to indicators of systemic inflammation such as circulating interleukin-1-beta (IL-1β), IL-6 and tumor necrosis factor-alpha (TNF-α), and upregulated immune cell (leukocyte) gene expression for these proinflammatory cytokines and others. For instance, among breast cancer patients undergoing primary treatment, greater depressive symptoms, negative affect, cancer-specific distress, and low social support have been related to greater serum IL-1β, IL-6, TNF-α, IL-1 receptor antagonist (IL-1RA), and TNF receptor II (TNF-RII) levels (Blomberg et al. 2009, Bouchard et al. 2016, Bower et al. 2011); greater s100A8/A9 levels (Taub et al. 2019); greater leukocyte nuclear NFκB DNA binding (Diaz et al. 2021); and greater leukocyte *IL1A*, *IL1B*, *IL6*, and *TNFA* gene expression as well as increased expression of several chemokine, COX2 (prostaglandin-E, or *PGE*), and prometastatic (e.g., *MMP-9*) genes (Antoni et al. 2012, Jutagir et al. 2017).

With growing interest in the effects of stress factors on transcriptional (gene expression) changes in cancer and immune cells, molecular work has related stress-related variables to a comprehensive gene expression profile termed the conserved transcriptional response to adversity (CTRA) (Slavich & Cole 2013). The CTRA pattern describes the impact of stress responses to threats on immune system components originally developed to optimize survival. Accordingly, immune responses to threatening stressors were initially designed to optimize innate immunity (inflammatory reactions) against bacterial infections due to physical attack by directing energy away from antiviral (interferon-mediated) and antibody [immunoglobulin (Ig)-making] immune system components. This CTRA pattern is believed to have been conserved as a response to modern-day psychosocial stressors (Fredrickson et al. 2013, Slavich & Cole 2013). Using a CTRA index based on 51 inflammatory (e.g., greater proinflammatory cytokines, chemokines, and COX2), antiviral (e.g., lower IFN type I and type II), and antibody (e.g., lower Ig) genes, researchers have related greater leukocyte CTRA expression to psychosocial adversity conditions such as greater negative affect, depressive symptoms, and lower socioeconomic status (SES) (Cohen et al. 2012, Knight et al. 2016) in cancer patients.

### Psychological and Physiological Adaptation and Clinical Course of Cancer

**Figure 1:**
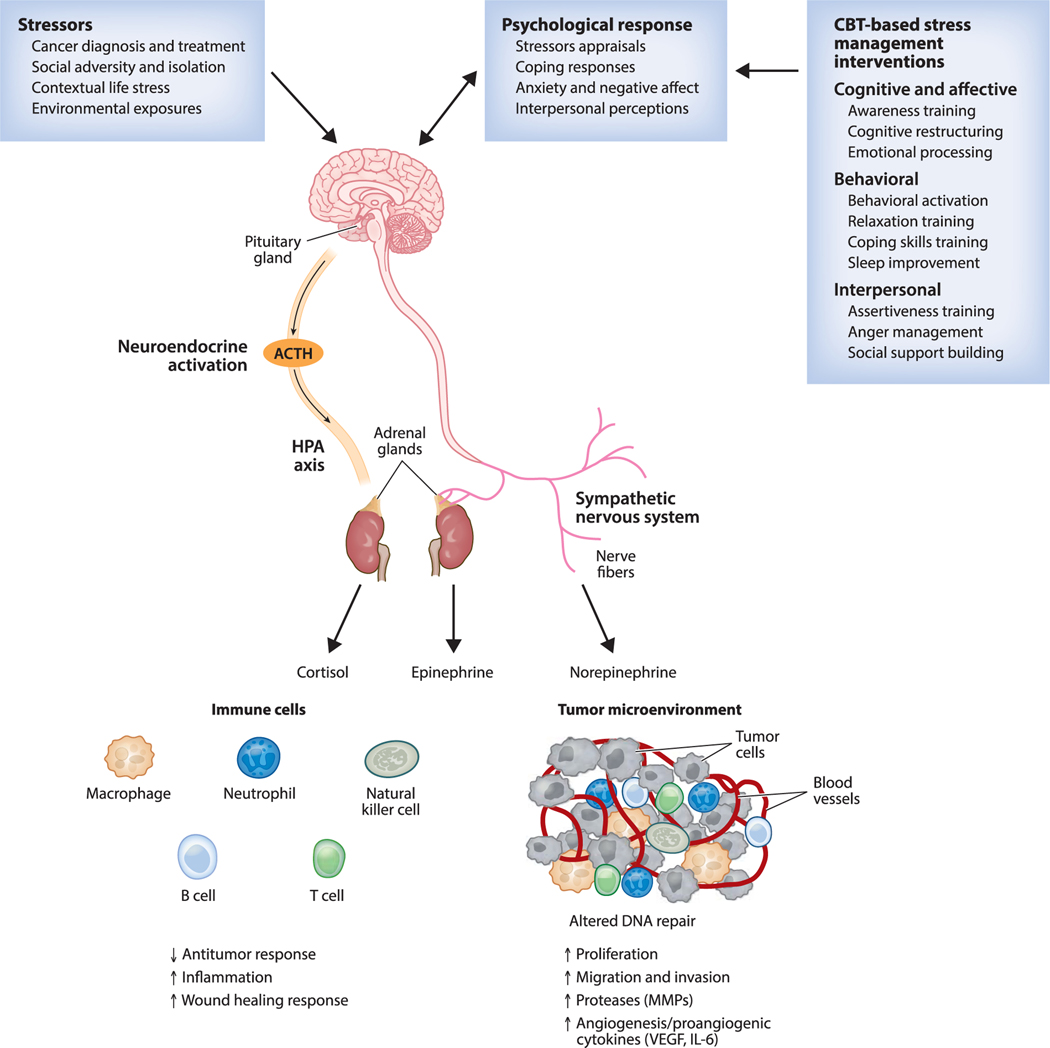
Biobehavioral model for stressors, psychological responses, neuroendocrine activity, and impact on peripheral tissue in cancer and their interactions with cognitive behavioral therapy (CBT)-based stress management interventions. Abbreviations: ACTH, adrenocorticotrophic hormone; HPA, hypothalamic-pituitary-adrenal; IL, interleukin; MMP, matrix metalloprotease; VEGF, vascular endothelial growth factor.

There is growing evidence that adverse psychosocial factors (depression, distress, low social support, low SES) are associated with shorter survival time for a wide number of different cancers (Chida et al. 2008). For instance, greater depressive symptoms predict shorter overall survival in patients treated for nonmetastatic (Antoni et al. 2017) and metastatic (Giese-Davis et al. 2011) breast cancer and in patients with RCC (Cohen et al. 2012). Lower SES predicts shorter leukemiafree survival (Knight et al. 2016), and lower social support predicts shorter survival in patients with ovarian cancer (Lutgendorf et al. 2012). As noted previously, there are several comprehensive reviews of the neuroendocrine pathways underlying physiological stress responses and their associations with important biological processes that promote disease progression (Antoni & Dhabhar 2019, Antoni et al. 2006b, Chang et al. 2022, Eckerling et al. 2021). This literature provides a rationale for investigating the effects of stress management interventions (SMIs) to optimize health in cancer patients through their role in modulating biobehavioral processes. Figure 1 summarizes our contemporary understanding of a biobehavioral model for the role of stressors, psychological responses, and neuroendocrine activity on peripheral tissue (immune and cancer cells) and the putative role of cognitive behavioral therapy (CBT)-based SMIs in modulating these processes in cancer patients.

## IMPACT OF STRESS MANAGEMENT INTERVENTIONS ON PSYCHOLOGICAL ADAPTATION IN CANCER PATIENTS AND SURVIVORS

### What Constitutes a Stress Management Intervention?

Since modifying responses to stressors might improve adaptation to cancer, QoL, and health outcomes, several randomized controlled trials (RCTs) have tested interventions that can be considered SMIs in the past 50 years (Antoni & Dhabhar 2019). Some SMIs tested in cancer patients work by reducing tension in order to decrease physiological activation through physical techniques such as relaxation (typically muscle relaxation training and deep breathing), Yoga and Tai-Chi, massage, acupuncture, and biofield therapies (Antoni & Dhabhar 2019). Other SMI approaches focus on increasing awareness and developing a nonjudgmental attitude about ongoing stressors and stress appraisals via mindfulness meditation techniques (Antoni & Dhabhar 2019). A final set of SMIs work by teaching skills for modifying cognitive appraisals of stressors and developing new coping strategies through CBT techniques, such as cognitive restructuring and coping effectiveness training, and by building interpersonal/communications skills to better access and maintain coping resources such as social support (Antoni & Dhabhar 2019). CBT-based approaches will be the chief focus of this review.

It is noteworthy that other psychosocial/behavioral interventions that are not necessarily SMIs have shown efficacy in cancer populations. These include supportive expressive therapy (SET) (Spiegel et al. 1989) targeting existential issues; palliative care interventions targeting symptom and pain management (Temel et al. 2010); and physical exercise interventions targeting physical activity, strength, and aerobic fitness (McNeely et al. 2006). Because the effects of physical-based and mindfulness-based SMIs in cancer patients are covered in a separate review in this volume (Carlson 2023), we will focus mostly on the CBT-based SMI approaches (which often include CBT and relaxation techniques) and their effects on psychological adaptation, physiological adaptation, and clinical health outcomes. Interested readers are also referred to other recent narrative and quantitative reviews that summarize the biological and clinical health effects of some of these other intervention approaches in cancer patients and survivors (Chang et al. 2022, Eckerling et al. 2021, Mirosevic et al. 2019).

### Cognitive Behavioral Therapy as a Stress Management Intervention to Facilitate Psychological Adaptation

CBT refers to a class of interventions that share three fundamental principles: Cognitions affect behavior, cognitive patterns can be monitored and changed, and desired change in behavior can be achieved through change in cognitions (Dobson & Dozois 2010). Specific CBT interventions vary in the degree to which they focus on cognitive change versus directly targeting behavioral change. In addition to its focus on cognitive and behavioral change, CBT also addresses the bidirectional impact of cognitions and behavior on both emotions and physiology. CBT interventions are typically brief, goal-oriented, and based on principles of learning and behavior change and aim to reduce symptoms, improve functioning, and remit psychiatric disorders (Hofmann et al. 2012). Examples of CBT intervention strategies include behavioral activation, cognitive restructuring, relaxation training, biofeedback, guided imagery, problem solving, assertiveness and communication training, contingency management, and systematic desensitization. CBT is one of the most widely studied intervention approaches and has strong evidence for effectiveness in several disorders and symptom clusters that are common in the cancer context, including anxiety, depression, and overall psychological distress and stress (Hofmann et al. 2012).

### Effects of Cognitive Behavioral Therapy Interventions on Psychological Adaptation in Cancer Patients and Survivors

Research examining the effects of CBT on individuals affected by cancer has flourished. Meta-analytic evidence demonstrates that CBT interventions support psychological adaptation by reducing anxiety, depressive symptoms, and both general and cancer-specific emotional distress as well as by improving emotional well-being and overall QoL in both individuals diagnosed with cancer and their caregivers (Cobeanu & David 2018, Getu et al. 2021, Hart et al. 2012, Osborn et al. 2006, O’Toole et al. 2017, Tatrow & Montgomery 2006). CBT interventions also facilitate interpersonal adaptation and relationships with close others by improving social support, communication, sexual functioning, and overall relationship quality and satisfaction (O’Toole et al. 2017). With respect to symptom burden, CBT interventions reduce pain, sleep disturbance, fatigue, and treatment side effects like nausea and vomiting (Cobeanu & David 2018, Getu et al. 2021, Johnson et al. 2016, Sheinfeld Gorin et al. 2012, Tatrow & Montgomery 2006).

Nevertheless, research on the effects of CBT includes mixed findings. Observed effect sizes range from small to large (Cobeanu & David 2018, Osborn et al. 2006, O’Toole et al. 2017), and there are null findings for pain, physical functioning, overall QoL, and targets of intervention such as coping skills and self-efficacy (Osborn et al. 2006, O’Toole et al. 2017). Mixed findings may be due to significant variability in methodological design such as face-to-face versus online/telephone delivery, individual- versus group-based intervention, duration and number of sessions, and active versus nonactive controls. Furthermore, eligibility criteria vary by cancer type, stage of diagnosis, time since diagnosis, and active treatment versus post-treatment completion/surveillance. Some evidence suggests that women and younger individuals may benefit more from CBT interventions (O’Toole et al. 2017) and individual-based interventions may be superior to group-based interventions (Cobeanu & David 2018, Tatrow & Montgomery 2006). In contrast, other reviews have suggested that older and unpartnered cancer patients and those with earlier-stage disease may show greater effects of CBT-based SMIs on long-term health outcomes (Mirosevic et al. 2019). However, analyses examining whether design characteristics moderate the effects of CBT have yielded few significant results, and more research is needed (Cobeanu & David 2018, Getu et al. 2021, O’Toole et al. 2017).

Studies of CBT interventions in the cancer context have largely focused on women with early-stage breast cancer (Cobeanu & David 2018, Getu et al. 2021, Tatrow & Montgomery 2006). Importantly, few studies select for cancer patients and survivors with baseline elevations in distress and symptom burden, despite growing meta-analytic evidence suggesting that these individuals benefit most from psychological interventions (Heron-Speirs et al. 2012, Schneider et al. 2009, Sheard & Maguire 1999). It is difficult to demonstrate the benefit of CBT in individuals with low levels of baseline distress or symptom burden as outcomes of interest have little room for improvement (Stanton 2005). One meta-analysis demonstrated that CBT decreases symptoms of depression among individuals with cancer who had elevated symptoms at baseline with effect sizes that were significantly larger than those of problem-solving therapy and marginally larger than those of pharmacologic interventions (Hart et al. 2012). Therefore, more research is needed to understand the impact of CBT interventions among individuals with a diversity of cancers, including rare cancers and advanced/metastatic cancers. Some CBT-basedinterventionshavebeen explicitly developed to serve as SMIs.

### Cognitive Behavioral Stress Management Effects on Psychological Adaptation in Cancer Patients and Survivors

Cognitive behavioral stress management (CBSM) is a 10-week CBT-based SMI that incorporates cognitive, behavioral, and interpersonal skills training and relaxation training through in-session didactic and role-playing activities as well as homework and daily practice to help improve QoL and reduce symptoms (Antoni 2003a, Penedo et al. 2008). This protocol integrates core CBT principles and practices such as cognitive restructuring (identifying and disputing irrational or maladaptive thoughts), behavioral activation (engagement in pleasant experiences, social activity, or experiences of mastery), and relaxation training like diaphragmatic breathing, progressive muscle relaxation, and meditation/imagery. Example CBSM intervention topics include introducing stress awareness and physical responses, stress awareness and the appraisal process, automatic thoughts and cognitive distortions, cognitive restructuring and rational thought replacement, coping strategies, social support, anger management, and assertiveness training. Research demonstrates that CBSM confers numerous effects on psychological adaptation in cancer survivors, including improved overall QoL and social support, increased positive affect, benefit finding, and relaxation and coping skills as well as reduced depressive symptoms, anxiety, and emotional distress (Addison et al. 2022; Antoni et al. 2006a,c, 2009; Penedo et al. 2004, 2006; Tang et al. 2020).

## IMPACT OF STRESS MANAGEMENT INTERVENTIONS ON PHYSIOLOGICAL ADAPTATION AND HEALTH OUTCOMES IN CANCER PATIENTS

### Methodologic Considerations

It is challenging to use psychological interventions as a paradigm to test the impact of stress reduction on psychological adaptation to cancer and its treatment, on changes in stress physiology, and on long-term clinical outcomes and survival (Antoni & Dhabhar 2019). This requires demonstrating that a specific SMI can (*a*) modulate psychological adaptation indicators (e.g., stress management skill efficacy, reduced distress, anxiety, depression) in cancer patients in tandem with (*b*) changes in neuroendocrine indicators (decreased or normalized SNS and HPA axis activity), (*c*) immune measures (decreased circulating inflammatory markers and inflammatory signaling in cells, and increased cellular immune function), and (*d*) long-term effects on QoL and physical health status (cancer recurrence, survival/mortality) (Antoni & Dhabhar 2019). Such studies are difficult because they require recruiting patients into an RCT at a specific point in treatment (e.g., at diagnosis, pre- or post-surgery, during primary treatment, or at the time of disease recurrence); inducing improvements in psychological (distress) and physiological (neuroendocrine and immune parameters) adaptation via SMI across the initial period of treatment and into survivorship; and following cohorts for several years for clinical outcomes. We first present evidence that various CBT-based SMIs shown to improve psychological adaptation also show salutary effects on physiological adaptation (neuroendocrine and/or immune system variables) in cancer patients. We then highlight research on a couple of SMIs showing long-term clinical benefits that may be explained by earlier physiological changes. Finally, we compare and contrast these interventions and the study designs used to demonstrate these effects.

### Effects of Stress Management Interventions on Physiological Adaptation in Cancer Patients and Survivors

The RCTs showing neuroendocrine and/or immune system effects of SMIs on cancer patients have been limited to trials enrolling nonmetastatic cancer patients, including patients with breast cancer and malignant melanoma, at the early stages of diseases. Generally, studies showing intervention effects on physiological adaptation in cancer patients also showed parallel effects on psychological adaptation (McGregor & Antoni 2009). An early study demonstrating the effects of SMIs on immune indices was an RCT showing that stage 1–2 malignant melanoma patients assigned to a 6-week group CBT-based SMI (coping skills training, relaxation) improved psychological adaptation (negative mood) at 6weeks (Fawzy et al. 1990a) and cellular immune functioning (NKCC) at 6 months (Fawzy et al. 1990a). This trial showed that SMIs can improve indices of cellular immunity in patients who are receiving only surgical intervention. Can SMIs help promote recovery or preservation of immune functioning as patients go through the storm of adjuvant therapies?

A 12-month group-based SMI that included relaxation and CBT-based stress reduction exercises, coping skills training, and health education in 14 weekly sessions and 8 monthly maintenance sessions decreased distress and negative health behaviors (eating high-fat foods and tobacco smoking) over the initial 4 months, and it increased cellular immunity [lymphocyte proliferative response (LPR)] compared to treatment as usual in stage 2–3 breast cancer patients recruited in the period after surgery (Andersen et al. 2004).

Similarly, a 10-week group CBSM intervention (Antoni 2003b) was tested in stage 0–3 breast cancer patients at a similar point in their treatment (2–10 weeks post-surgery and prior to initiating adjuvant chemotherapy or radiation). In two separate RCTs, compared to a 1-day psychoeducational (PE) control, CBSM was shown not only to improve cancer-specific distress, mood, social adversity, and QoL (Antoni et al. 2001, 2006a,c) but also to decrease afternoon-evening (PM) serum cortisol levels (Phillips et al. 2008) and increase LPR and IL-2 and IFN-γ production (Antoni et al. 2009, McGregor et al. 2004). This is one of the only SMIs showing reductions in serum cortisol in two separate trials (Cruess et al. 2000, Phillips et al. 2008). Showing reductions in evening cortisol is relevant because flatter diurnal cortisol slopes (due partly to higher PM levels) have been associated with several cancer-promoting processes noted previously (Chang et al. 2022) and with decreased survival in breast cancer (Sephton et al. 2000), nonsmall cell lung cancer (Sephton et al. 2013), and RCC (Cohen et al. 2012). CBSM effects on LPR in one trial (McGregor et al. 2004) mirror those found in the prior 12-month CBT-based SMI by Andersen et al. (2004). CBSM effects on Th1 cytokine (IL-2 and IFN-γ) production in a second trial (Antoni et al. 2009) may be important for supporting cellular immune processes involved in tumor eradication as well as for promoting protection against opportunistic viral infections during adjuvant therapy.

CBSM was also shown to affect inflammatory signaling using leukocyte gene expression measures (Antoni et al. 2012). Those assigned to CBSM (versus PE control) showed altered expression of 91 leukocyte genes over the 6–12 months of treatment. These effects included downregulation of 62 genes for proinflammatory cytokines (*IL1B, IL6, TNF*), of inflammatory chemokines and their receptors (COX2/PGS2), and of mediators of tissue remodeling and EMT (e.g., *MMP-9*), together with upregulation of 29 genes related to cellular immune responding (type I IFN response, type II IFN signaling, and IFN signal transduction) (Antoni et al. 2012). Over 50% of the genes affected by CBSM were the same genes associated with negative affect at baseline. Since women assigned to CBSM showed significant concurrent reductions in negative affect and increases in positive affect, these transcriptional changes are likely attributable to the psychological adaptation improvements reported by women in CBSM. Bioinformatic analysis of this pattern of gene expression change inferred decreased NFκB/Rel and globin transcription factor (GATA) family activity and increased IFN response factors, all of which were linked to SNS signaling in prior work (Stark et al. 2001). Those in CBSM also showed increased expression of GR-related genes relative to controls and an overrepresentation of GR response elements in the promoters of CBSM-upregulated genes (Antoni et al. 2012). This provides compelling neuroimmune evidence that CBSM may reverse stress-induced GR desensitization (Miller et al. 2008), which could subsequently act to reduce inflammatory signaling (Miller et al. 2009). A reanalysis of this trial used gene expression results in a 51-gene CTRA composite score and noted that while breast cancer patients assigned to PE control showed marked increases in CTRA, those in CBSM showed slight decreases over 12 months of cancer treatment (Antoni et al. 2016). This pattern whereby CBSM mitigates a rise in CTRA signaling over the storm of adjuvant therapy (radiation and chemotherapy) has clinical significance in the context of ongoing cancer treatments, which are known to be proinflammatory.

### Effects of Stress Management Interventions on Physical Symptoms During and After Cancer Treatment

The literature documenting the effects of cognitive-based SMIs on physical health outcomes ranges from studies showing relatively short-term effects on physical symptoms during and after cancer treatment to those showing longer-term effects on clinical disease endpoints, such as disease-free interval (time till recurrence) and cancer-specific and overall survival. Among studies of physical symptoms during cancer treatment, some CBT-based SMIs have been associated with reductions in sleep disruption (Lechner et al. 2014, Savard et al. 2005), fatigue severity and fatigue-related disruption (McFarlandet al. 2021, Vargas et al. 2014), and pain (Key et al. 2021) and improved sexual and urinary functioning (Molton et al. 2008). CBT-based SMIs have also been associated with improvements in general health ratings by staff and patient-reported outcomes of health-related QoL and well-being. For instance, stage 2–3 breast cancer patients undergoing treatment assigned to a 12-month CBT-based SMI showed better health status based on staff ratings at 12-month follow-up, and initial reductions in distress at 4 months predicted better health status at 12 months (Andersen et al. 2007a). Breast cancer patients assigned to a 10-week CBSM intervention reported improved fatigue-related daily dysfunction and sleep quality, less difficulty falling asleep, and reductions in onset insomnia over 12 months, with sleep latency effects mediated by increased perceived stress management skills (Vargas et al. 2014). Greater improvements in sleep quality also predicted greater reductions in fatigue-related daily dysfunction over 12 months.

Men treated for prostate cancer report significant physical side effects, including sexual and urinary dysfunction, fatigue, and pain, along with anxiety and depressive symptoms, decreased QoL, and threats to masculine identity (Bennett & Badger 2005, Lintz et al. 2003). Among men treated for early-stage prostate cancer, a 10-week CBSM (versus a 1-day PE control) improved QoL (Penedo et al. 2004) and sexual functioning pre/post intervention (Molton et al. 2008), and CBSM effects on QoL were mediated by improved perceived stress management skills (Penedo et al. 2006). Men entering the intervention with higher levels of anxiety or interpersonal dysfunction (e.g., hostility, interpersonal sensitivity) derived greater benefit from the intervention, as evidenced by greater improvements in sexual and urinary function, relative to men with lower levels of these traits. A Spanish-language version of CBSM with some attention to cultural factors (e.g., family interdependence) improved sexual function and physical, emotional, and total well-being in monolingual Hispanic men treated for prostate cancer (Penedo et al. 2007). Although most studies of CBT-based SMIs have been restricted to patients with breast or prostate cancer, there is evidence that CBT-based approaches may mitigate physical symptoms in other types of cancer during treatment and into survivorship (Breitbart et al. 2021).

### Effects of Psychological Intervention on Long-Term Clinical Outcomes in Cancer Survivors: Initial Studies

The question of whether psychosocial interventions can improve long-term clinical outcomes in cancer patients has been of longstanding interest and a source of controversy in the field since the report by Spiegel et al. (1989) that metastatic breast cancer patients randomized to a 12-month group-based SET intervention appeared to live twice as long (~36 months) as those assigned to treatment as usual (~18 months). This report was a major driver of RCTs over the next 30 years testing a variety of different psychosocial interventions for survival effects. Efforts to replicate these effects in metastatic breast cancer patients have been unsuccessful to date in larger samples using the same SET intervention protocol (Goodwin et al. 2001, Spiegel et al. 2007), though subgroups of patients with a poorer prognosis (those with estrogen receptor–negative disease) have shown improved survival with SET (Spiegel et al. 2007). However, other trials testing CBTbased and SET-based interventions in patients with metastatic breast cancer have also failed to show effects on overall survival (Kissane et al. 2007).

In one of the first trials to report the effects of a CBT-based SMI on psychological, biological, and health outcome parameters, Fawzy and colleagues observed among 66 patients with malignant melanoma that those assigned to a 6-week group intervention showed improved coping and mood (Fawzy et al.1990a), increases in NKCC (Fawzy et al. 1990b), and longer survival and lower odds of recurrence over a 6-year follow-up (Fawzy et al. 1993), but these effects on survival were no longer significant at 10 years (Fawzy et al. 2003). Unfortunately, it does not appear that this group reported associations between intervention-related biological changes and long-term clinical outcomes.

### Meta-Analyses of Effects of Stress Management Interventions on Long-Term Survival in Cancer

Looking across the entire psycho-oncology literature, several reviews reported primary or secondary analyses of RCTs for which follow-up data on clinical endpoints were available for periods ranging from 1 to 15 years. Efforts to summarize this literature have appeared in multiple qualitative and quantitative reviews (and at least 8 meta-analyses) in the past 10 years (Antoni 2013, Eckerling et al. 2021, Mirosevic et al. 2019, Oh et al. 2016). A meta-analysis of 15 RCTs completed prior to 2015 and meeting Cochrane criteria for methodological quality involved nearly 3,000 cancer patients (Oh et al. 2016). Results indicated no overall survival benefits of a variety of psychosocial interventions; however, interventions delivered early in disease (in 6 trials with 1,448 patients with nonmetastatic disease) showed a 41% reduced risk of cancer mortality (Oh et al. 2016). Since the time of this meta-analysis, other major reviews and meta-analyses have generally supported the notion that SMIs may show significant effects on overall survival in cancer patients (Eckerling et al. 2021, Mirosevic et al. 2019). These later reviews are based on trials published up to 2017, include patients with multiple cancer types and disease stages, and focus on interventions of various theoretical orientations (e.g., CBT, SET) and delivery formats (individual, group).

One recent review focused on 22 studies reporting long-term effects of what were referred to as stress-reducing interventions among patients with nonmetastatic breast cancer (N = 5), metastatic breast cancer (N = 7), malignant melanoma (N = 2), and several other cancer types (N = 8) including lymphoma; esophageal, lung, and gastrointestinal cancer; and samples of mixed cancer types (Eckerling et al. 2021). These trials were quite heterogeneous regarding sample size (N = 60–303), cancer type and stage, treatment orientation, individual versus group delivery format, duration, and timing within the cancer care continuum. Eckerling et al. (2021) noted that of the 22 studies examined, 8 reported a significant survival effect. Among breast cancer patients, the two trials showing survival benefits for patients with nonmetastatic disease were CBT-based group SMIs with 11-year follow-up periods (Andersen et al. 2008, Stagl et al. 2015b), and the one showing survival benefits for metastatic breast cancer was group SET with a 10-year follow-up (Spiegel et al. 1989). Among interventions for malignant melanoma, CBT-based SMIs showed survival effects in one trial over a 6-year follow-up (Fawzy et al. 1993) but not in another trial with a 4- to 6-year follow-up (Boesen et al. 2011).

A more selective meta-analysis included 12 trials (N = 2,439 patients) drawn from the Cochrane Central Registry of Controlled Trials, Ovid MEDLINE, PubMed, PsycINFO, and prior meta-analyses for the period 1970–2017. Included trials met several a-priori selection criteria [e.g., randomization, sufficient number of events (>10% deaths), use of intent-to-treat (ITT) analysis] (Mirosevic et al. 2019). The meta-analysis reported an overall significant but small-to-moderate effect of psychosocial interventions on survival from study entry until death [mortality hazard ratio (HR) = 0.71; 95% confidence interval (CI) = 0.58–0.88; *p* < 0.002] (Mirosevic et al. 2019). Among all psychological interventions examined, the effects were stronger in persons who were not partnered (*p* < 0.005) and in older (≥60 years) versus younger (<50 years) patients (*p* < 0.01) (Mirosevic et al. 2019). Of interest for the present review, they also reported that for CBT-based interventions, the treatment arm was more likely to show significant effects on overall survival in cancer patients recruited at earlier stages of disease (early HR = 0.30 versus later stage HR = 1.1; *p* = 0.01 for difference). In fact, the two studies showing the largest effects on survival were both group CBT-based SMIs in nonmetastatic breast cancer patients followed over an 11-year median. Andersen et al. (2008) reported HR = 0.44, 95% CI = 0.22–0.86; Stagl et al. (2015b) reported HR = 0.21, 95% CI = 0.05–0.93; and both studies reported large effect sizes. In terms of clinical significance, these two trials yielded number needed to treat (NNT) values of 1.53 (Stagl et al. 2015b) and 2.92 (Andersen et al. 2008), with NNT ≤ 3 considered a large effect (Citrome 2008). Since these two trials accounted for 467/2439 (20%) of the cases analyzed in the meta-analysis, we now review them in further detail.

### Exemplars of Modern Trials That Demonstrate the Effects of Stress Management Interventions on Long-Term Clinical Health Outcomes in Breast Cancer Patients

We now focus on the two trials showing CBT-based SMI effects on psychological adaptation, physiological adaptation, and long-term clinical health outcomes within the same trial, and we examine the evidence showing that changes in physiological adaptation predict or mediate the magnitude of the interventions’ effects on clinical outcomes measured at up to 15 years into survivorship. This affords the opportunity to study the interventions’ effects not only on overall and cancer-specific survival but also on disease-free interval—that is, the period of time patients remain free of a recurrence of primary disease or a secondary cancer.

Andersen et al. (2008) reported that among 227 nonmetastatic stage 2–3 breast cancer patients, those assigned to the 12-month group CBT-based SMI described previously (Andersen et al. 2004), followed for 8–13 years, showed significantly greater 11-year median overall and breast cancer–specific survival rates as well as a 45% reduced risk of cancer recurrence compared to those assigned to treatment as usual. In a subgroup of depressed women in this cohort monitored over this follow-up period, those receiving the intervention showed decreases in total white blood cells (WBC) and neutrophils (changes consistent with less systemic inflammatory signaling) compared to controls (Thornton et al. 2009). Importantly, women whose cancer recurred revealed greater serum cortisol and inflammation (greater total WBC and neutrophils) 17 months prior to their recurrence (Thornton et al. 2008). Moreover, those who experienced a distal recurrence had weaker cellular immune responses (LPR, NKCC) and greater elevations in WBC compared to those experiencing only a local recurrence (Thornton et al. 2008). During the 12 months following recurrence, the intervention group also showed improved psychological adaptation (decreased negative mood and increased social support) and physiological adaptation (greater LPR and NKCC), suggesting some protection against stress-related biological changes during the challenges of further treatments for their disease progression. Finally, women who had received the intervention previously had a lower risk of death over the subsequent 80 months postrecurrence compared to controls (Andersen et al. 2010). Taken together, these analyses support the hypothesis that a CBT-based SMI that improves psychological adaptation (i.e., decreases distress) may maintain better physiological adaptation during the disease-free years of survivorship, reduce the odds of mortality and recurrence, and promote persisting benefits even after disease recurrence.

Another RCT reported CBT-based SMI effects on psychological adaptation (Antoni et al. 2006c), physiological adaptation (Antoni et al. 2009, Phillips et al. 2008), and long-term clinical health endpoints (Stagl et al. 2015b). Among 240 women with stage 0–3 breast cancer, those assigned to a 10-week CBSM group intervention showed lower odds of mortality and recurrence at 8–15-year (11-year median) follow-up over and above the effect of age, time since surgery, stage, tumor receptor type (estrogen receptor/progesterone receptor), tumor size, and adjuvant therapy (Stagl et al. 2015b). In analyses of patients matched for the same stage (stage 2–3) as the patients enrolled in Andersen et al.’s (2008) trial, CBSM showed even larger reductions in odds of breast cancer mortality and recurrence (Stagl et al. 2015b). It is noteworthy that survivors in this cohort followed with self-report measures over 8–15 years also reported significantly lower depression and better QoL at 11-year median follow-up compared to controls (Stagl et al. 2015a). This suggests that intervention effects were durable and remained in place over the period of monitoring long-term health outcomes. Investigators then tested whether changes in physiological adaptation via CBSM could explain its effects on disease-free survival (DFS). This is because most biobehavioral models of stress and cancer course propose that stress-induced neuroendocrine effects on immune and tumor cell signaling may enhance the odds of metastatic spread and disease recurrence (e.g., via angiogenesis, invasion, EMT, anoikis) (for reviews, see Antoni & Dhabhar 2019, Antoni et al. 2006b, Chang et al. 2022, Cole et al. 2015,  Eckerling et al. 2021). Since Andersen and colleagues’ RCT had implicated inflammation and WBC recruitment in predicting breast cancer recurrences (Thornton et al. 2008, 2009), investigators tested whether CBSM-induced changes in circulating leukocyte transcriptional activities during initial treatment explained the effects of CBSM on increased 11-year DFS in this cohort. To do so, they used the previously described CTRA gene expression composite (Fredrickson et al. 2013) at baseline, 6 months, and 12 months. Patients assigned to the control showed significant increases in CTRA in their leukocytes over the 12 months of the trial, while those assigned to CBSM showed small decreases; a lower CTRA increase over the 12 months of initial treatment predicted greater 11-year DFS (Antoni et al. 2016). Interestingly, most recurrences had taken place by the 8-year follow-up, and about 80% of women classified in the low CTRA change group remained disease free over this time compared to only 20% of those in the high CTRA increase group, even after controlling for age, stage, and chemotherapy, radiation, and endocrine therapy receipt (DFS HR = 6.32; 95% CI = 1.41–28.34) (Antoni et al. 2016).

It is plausible that adjuvant therapy receipt increases CTRA signaling and that this is compounded by chronic stress during initial treatment. If so, CBSM may mitigate the contribution of chronic stress to CTRA, and maintaining lower CTRA over this cancer treatment period may decrease the odds of pro-metastatic signaling and thereby improve long-term DFS (Antoni et al. 2016). This explanation of the effects of CBSM—and possibly other SMIs (e.g., Andersen et al. 2008)—on long-term health outcomes in breast cancer patients implies that when delivered during initial treatment, SMIs work by mitigating the compounding effects of stress on an already biologically adverse milieu. Specifically, CBT-based SMIs might improve long-term health outcomes in breast cancer patients by modulating the activities of immune cells (e.g., inflammation), which have the potential to communicate with cancer cells and other immune cells in the tumor microenvironment to promote metastasis (Antoni & Dhabhar 2019, Chang et al. 2022). This may also have implications for SMIs in other cancers, since greater expression of this same combination of genes (i.e., CTRA) also predicts increased relapse risk and decreased leukemia-free survival in recipients of hematopoietic stem cell transplant (HSCT) for acute myelogenous leukemia (Knight et al. 2016).

Which aspects of these two comprehensive SMI trials of breast cancer patients can provide guideposts for future mechanistic work in the field? Although conducted by different laboratories, the two CBT-based SMI RCTs in nonmetastatic breast cancer patients reviewed here are similar in terms of sample size (N = 227 and N = 240), timing (>2 weeks post-surgery and prior to starting adjuvant therapy), intervention format (group), theoretical orientation (CBT-based SMI), frequency (weekly for initial training over 10–14 weeks), and follow-up intervals for main outcomes (psychological and immune measures over 12 months, clinical disease outcomes over a median of 11 years). A deeper look at each trial reveals that reported distress and/or cortisol decreases were related to either increased frequency of relaxation practice or increased confidence in using SMI skills such as relaxation and cognitive restructuring (Andersen et al. 2007b, Phillips et al. 2011). Although the disease stage range of the samples did differ in the two trials [stage 2–3 in Andersen et al.’s (2008), stage 0–3 in Stagl et al.’s (2015b)], women in the SMI conditions had similar reduced odds of a recurrence compared to controls [HR = 0.55 in Andersen et al.’s (2008), HR = 0.45 in Stagl et al.’s (2015b)]. When analyses were restricted to stage 2–3 cases only, intervention effects on recurrence were somewhat higher in CBSM. A similar pattern was evident for all-cause mortality and breast cancer–specific mortality, with larger impact for CBSM when restricted to stage 2–3 cases. This suggests that although CBT-based SMIs appear more effective in nonmetastatic cases (stage 0–3) on meta-analyses (Mirosevic et al. 2019), their effect on clinical health outcomes may actually be strongest in women with mid-stage disease (stage 2–3), where the odds of metastasis and mortality are higher than in earlier stages (stage 0–1).

Differences in the two trials include a longer period of continuous intervention [12 months in Andersen et al.’s (2008) versus 10 weeks in Stagl et al.’s (2015b)] and an additional focus on health behavior change in one intervention (Andersen et al. 2008). Comparing trial results suggests that it may be plausible to produce long-term health benefits in the briefer program with a sole focus on SMI skills training.This raises the question of whether an even briefer SMI could be enough to modulate similar biological pathways (i.e., inflammation) by modifying psychological adaptation in cancer patients undergoing treatment. This is also relevant for implementation considerations, since even a 10-week intervention might be challenging to deliver in clinical oncology settings. Because each intervention uses a combination of elements, including relaxation training, various CBT techniques, and also health education in one intervention (Andersen et al. 2008), it is important to understand which elements are accounting for these salutary effects.

A dismantling trial subsequently compared the psychological and physiological adaptation effects of three time- and attention-matched group interventions: 5-week relaxation training versus 5-week CBT versus 5-week health education in postsurgical breast cancer patients. Those assigned to either relaxation training or CBT showed improved mood and emotional well-being compared to those in health education, with similar effects in CBT and relaxation training conditions (Gudenkauf et al. 2015). Importantly, women assigned to either CBT or relaxation training also showed lower increases in inflammatory signaling (circulating s100A8/A9 levels; Taub et al. 2019) and NFκB DNA binding (Diaz et al. 2021) over 12 months compared to those in health education, with results similar in CBT and relaxation training. Specifically, women in health education showed significant increases in s100A8/A9 levels across the period of cancer treatment, while those in relaxation training or CBT showed slight declines (Taub et al. 2019). A separate study conducting intensive molecular analyses on women drawn from the same trial who had elevated cancer-specific distress at study entry examined changes in leukocyte cell nuclei over 12 months (Diaz et al. 2021). Again, women in health education revealed significant increases in nuclear NFκB DNA binding over 12 months, while those in CBT or relaxation training revealed small decreases over the same period (Diaz et al. 2021). These intervention effects on s100A8/A9 and NFκB DNA binding mirror a similar pattern observed in leukocyte proinflammatory gene expression in women with breast cancer participating in the trial of 10-week CBSM, who showed mitigation of the rise in CTRA (which includes five genes relevant for NFκB binding: *NFKB1*, *NFKB2*, *REL*, *RELA*, and *RELB*) (Antoni et al. 2016).

In terms of intervention-related processes, women receiving these brief SMIs showing the greatest increases in perceived stress management skills (i.e., relaxation, cognitive restructuring) pre/post intervention showed the lowest s100A8/A9 levels and NFκB binding over 12 months (Diaz et al. 2021, Taub et al. 2019). Less cancer-specific distress (i.e., intrusive thoughts) and noncancer-specific negative affect at 12 months are related to less NFκB binding (Diaz et al. 2021). This strongly implicates that changes in stress management processes and in psychological adaptation can account for these biological changes. Since all three interventions were 5 weeks long and group based, the differential effects of SMI (CBT or relaxation training) versus health education are likely due to stress management skills training rather than attention or group support, though the interactive effects of SMI training plus group support cannot be separated out. These findings may be clinically relevant, since s100A8/A9 levels have been shown to predict breast cancer metastasis (Kwak et al. 2017), and greater NFκB nuclear binding may enhance inflammatory gene expression underlying the CTRA pattern, which was shown to predict disease-free survival in breast cancer patients (Antoni et al. 2016). This cohort is currently being followed to assess the long-term clinical impact of these brief SMIs.

## EMERGING TOPICS IN STRESS MANAGEMENT RESEARCH IN CANCER

With growing evidence that CBT-based SMIs can help cancer patients manage the stress of treatment to improve psychological and physiological adaptation and clinical health outcomes, several new research foci are emerging in the field. We highlight a number of these in the Future Issues section. We end here with a summary of four issues that may be especially salient for moving forward the research on SMI in cancer in the coming decade.

### Role of Central Nervous System Processes in Research on Stress Management Interventions

First, we lack an understanding of the brain activities related to stress processing in cancer patients that could inform development of more precise SMI approaches. Reviews of the brain imaging literature have identified some key cortical and subcortical regions whose activity relates to individual differences in depressive symptoms, anxiety, and distress levels in cancer patients; these reviews have proposed the interoceptive network as a key network that should be included in future studies investigating brain-mediated biobehavioral processes in cancer (e.g., Reis et al. 2020). Greater distress/negative affect has been associated with less activity in cortical and subcortical regions that are important to stressor processing, including the anterior insula, thalamus, hypothalamus, ventromedial prefrontal cortex (PFC), and lateral PFC (Reis et al. 2020). To the extent that activity in the PFC and other regions is critical for optimal stress processing and stress management, this work suggests objective neural indicators that may be useful in future SMI research with cancer patients (Reis et al. 2020).

### Testing Pharmacologic Stress Management Interventions

Second, emerging work is using pharmacologic SMIs to directly modulate more peripheral stress-related neuroendocrine, immune, and tumor cell processes. To the extent that CBT-based SMIs may improve clinical outcomes in cancer patients by modulating biological processes, it is intriguing to ask whether other approaches could improve clinical outcomes by chemically modulating peripheral stress-associated pathways using pharmacologic interventions. One pharmacologic approach targets stress physiology pathways directly by using agents that antagonize SNS signaling (e.g., nonselective β-adrenergic blockade), whereas another approach uses anti-inflammatory agents (e.g., COX2 inhibitors) (for reviews, see Antoni et al. 2021, Chang et al. 2022). The ration ale for these approaches is based on preclinical work showing that NE and isoproterenol can enhance cancer-promoting processes (angiogenesis, anoikis), which are abrogated with β-adrenergic antagonists, and on clinical work showing that incidental use of β-blockers and COX2 inhibitors for other conditions is associated with reduced risk of cancer metastasis in humans (Antoni & Dhabhar 2019, Chang et al. 2022, Eckerling et al. 2021). For instance, the use of β-blockers is linked to reduced rates of progression (overall and disease-free survival) for solid (e.g., breast cancer, melanoma, colorectal cancer, lung cancer) and hematologic (e.g., multiple myeloma) malignancies (for reviews, see Chang et al. 2022, Eckerling et al. 2021). Future work might test whether a presurgical cocktail of a β-blocker (propranolol) and a COX2 inhibitor (etodolac), combined with brief CBT-based SMI in the postsurgical period, could provide an optimal regimen to facilitate the success of primary treatment for breast cancer and possibly other cancers (Antoni & Dhabhar 2019, Reis et al. 2020). There is also emerging work showing that stress-related changes in neuroendocrines may activate dormant cancer cells as well as interfere with the effectiveness of chemotherapeutic agents in controlling cancer (for a review, see Chang et al. 2022). This raises the provocative question, Could a combined pharmacologic and behavioral SMI regimen reduce the likelihood of stress-induced cancer cell activation and/or resistance to chemotherapy?

### Testing Effects of Stress Management Interventions on Long-Term Cancer-Associated Pathophysiologic Processes and Accelerated Aging

A third emerging area of research examines the effects of stress factors and SMIs on long-term cancer-associated pathophysiological processes (i.e., atherosclerosis, immune senescence) that accelerate mental and physical health decline in cancer survivors years after their treatments are completed [i.e., cancer accelerated aging (CAA)]. CAA is defined as the combined effects of cancer diagnosis, treatment, and their sequelae on the physiological aging process (Guida et al. 2019). Two areas of CAA in cancer survivorship include the role of the diminished immunoregulation in infectious disease (Dignani et al. 2014, Shehata & Karim 2014) and cardiovascular disease (CVD) (Mehta et al.2018). For example, immune responses to influenza vaccination in cancer patients receiving treatment are weakened (Shehata & Karim 2014), and influenza A is associated with a 66% greater incidence of pneumonia and an 18% mortality rate at 30 days in cancer patients (Dignani et al. 2014). Because stress factors can diminish the immune response to the influenza vaccine in older populations, and because inflammatory processes, which increase with age, stress, negative affect, and cancer treatment, diminish the vaccine response (Antoni & Dhabhar 2019), another needed application of SMI work is in the context of preventing opportunistic infections that occur during or after cancer treatment. This work might test whether SMIs improve the efficacy of protective vaccines in cancer survivors (e.g., Antoni et al. 2021).

Conditions associated with metabolic syndrome (i.e., dyslipidemia, hypertension, central obesity, and insulin resistance)—which represent major CVD risk factors—are often elevated in cancer survivors (Reis et al. 2020). The American Heart Association notes that CVD and associated risk factors (obesity and dyslipidemia) are increasing in breast cancer survivors (Mehta et al. 2018). Excessive weight is also associated with worse prognosis for breast cancer itself (Barone et al. 2021), with pre- and postmenopausal overweight and obese women having a greater likelihood of recurrence and mortality (Chan et al. 2014, Protani et al. 2010) and of developing a secondary cancer in the unaffected breast or at a separate primary site (Majed et al. 2011); these effects are all believed to be mediated, in part, by heightened inflammatory signaling. Recent work suggests that brief SMIs reduced serum IL-6 levels in obese and overweight breast cancer patients undergoing initial treatment (Ream et al. 2022). Large-scale trials should test the efficacy of SMIs for optimizing longer-term cardiovascular health and other consequences of CAA via improved immune regulation in this vulnerable population of cancer survivors.

### Extending the Reach of Research on Stress Management Interventions to Underserved Cancer Populations

A fourth area involves extending the reach of SMIs to the underserved through the use of culturally adapted interventions (Lechner et al. 2014, Penedo et al. 2018) and remote technologies (Penedo et al. 2020b). As in the case of health care more broadly, there exist huge and entrenched cultural, ethnic, and racial disparities in cancer morbidity and mortality rates (Miller et al. 2017). Our lack of knowledge about the impact of SMIs in disparate groups is likely due to deficits in the appropriateness and accessibility of evidence-based SMIs for cancer patients in ethnic and racial minorities and other marginalized groups without easy access to major cancer centers. Work that uses geo-epidemiological methods is identifying how entrenched features in the neighborhoods and living conditions of racial/ethnic minority cancer patients in the United States (i.e., structural racism; Bailey et al. 2021) appear linked to poorer breast cancer survival rates (Goel et al. 2022). How these structural or neighborhood-level factors can influence disease outcomes may be explained in part by excess exposure to chronic stressors (environmental and personal) as well as limited access to care (i.e., greater distance to a cancer center could result in later presentation and limited treatment options) and multiple behavioral pathways (lifestyle behaviors, poor adherence to medications, etc.). Some of these may operate on cancer progression and poor clinical outcomes through stress-related biobehavioral pathways. While SMIs may address some of these stress processes, they are likely to be beneficial to these populations only if interventions are culturally adapted in ways that make them acceptable to users and are delivered in ways that can be deployed on a wider scale.

Attending in-person SMIs may present challenges to specific cancer populations who navigate health care appointments while maintaining employment and child care responsibilities, to those hesitant to attend structured groups in institutional settings due to medical mistrust, or to patients isolated for infection control following procedures such as hematological stem cell transplant. All of these challenges may be exacerbated in patients with low-income jobs and who lack the financial resources for child care and time off work. Technological innovations make it now possible to offer interventions remotely over digital platforms and embedded within health systems and patient portals (Penedo et al. 2020b). Recently completed RCTs have shown that web-based CBSM platforms can improve psychological adaptation in men with prostate cancer (Penedo et al. 2020a, Yanez et al. 2015). The potential value for remote delivery solutions has been accelerated by the coronavirus disease 2019 (COVID-19) pandemic, which has hastened the development, acceptance, and integration of tele-medicine and tele-health into the medical treatment settings (Doraiswamy et al. 2020). This presents an opportunity and a challenge to swiftly establish the efficacy and relative effect size (versus in-person approaches) of remotely delivered psychological interventions for cancer patients in noninferiority trials. While these remote delivery venues are beginning to show effects on psychological adaptation in cancer patients, less is known about the ability of remotely delivered versions to recapitulate the effects of empirically validated in-person SMIs on physiological adaptation and on long-term health outcomes in cancer patients as presented here. However, RCTs are underway to examine the impact of remote care on psychological and physiological adaptation in patients with breast cancer (Antoni et al. 2021) and prostate cancer (Penedo et al. 2018). Beyond these four major emerging areas of research, it is valuable to reflect on contextual factors and methodological challenges going forward.

### The COVID-19 Pandemic as a Model of Contextual Stressors in the Lives of Cancer Patients and Survivors

Cancer patients and survivors have been disproportionately and significantly impacted by the COVID-19 pandemic. Emerging studies have documented that the COVID-19 pandemic has further exacerbated common challenges to psychosocial adaptation following a cancer diagnosis and treatment. In the absence of major contextual stressors, survivors experience relatively high rates of psychosocial (e.g., anxiety, depression, loss of employment, financial toxicity, social isolation, role strain) and physical (e.g., preexisting health conditions, fatigue, pain, sleep disruption) concerns due to cancer and its treatment. Furthermore, COVID-19 mitigation strategies leading to social isolation from friends and family, limited leisure activities, financial strain, and fears and concerns over the physical health status of an already compromised population synergistically contribute to greater levels of psychosocial distress in cancer survivors (Kuderer et al. 2020, McGinty et al. 2020, Nicola et al. 2020, U.S. Census Bur. 2021). COVID-19-related discontinuity in care and disruptions to ongoing and follow-up treatment can also have negative psychosocial and physical health outcomes among individuals who may already be experiencing care fragmentation and less-than-optimal follow-up and surveillance. In fact, cancer-specific and all-cause mortality may even be affected by COVID-19 experiences and stress via risk behaviors (e.g., lack of physical activity, changes in lifestyle behaviors including poor sleep and nutrition, and poor treatment adherence/follow-up) (Williamson et al. 2020). SMIs are ideally poised to address the contextual stressors presented by the pandemic and to provide cancer patients and survivors with the necessary stress management tools to effectively navigate the multiple challenges that can have a detrimental impact on health-related QoL and health outcomes.

### Methodologic Challenges Going Forward

Multiple challenges remain in SMI research in cancer populations. Importantly, progress needs to be made in developing systems, methods, and incentives to identify and engage underrepresented populations, many of whom face extensive barriers to accessing health care, let alone research trials.These include individuals who are medically vulnerable, such as those who are older,are obese, or have significant comorbidities as well as individuals who are minoritized because of race, ethnicity, sexual orientation, gender identity, and so on. As described above, technology and cultural adaptation are two possible approaches to improving the reach of SMIs to diverse populations. Relatedly, more research is needed to optimize not only the timing of intervention (i.e., before, during, and after cancer treatment) but also the length, frequency, and delivery of intervention contact. Best practices for recruitment and retention of cancer patients and survivors in SMI research are also largely understudied. Importantly, innovative trial designs, such as just-in-time adaptive intervention (JITAI), multiphase optimization strategy (MOST), and sequential multiple assignment randomized trial (SMART) (Collins et al. 2007, Klasnja et al. 2015), as well as advancements in measurement and assessment such as computer adaptive tests (CATs) and ecological momentary assessments (EMAs), have the potential to generate novel findings that inform future SMI research.

## CONCLUSION

With growing evidence for the efficacy of SMIs in cancer patients, future research questions will need to ask which, when, where, and for whom these interventions might be used optimally in clinical oncology settings (Antoni & Dhabhar 2019). The “which” question asks, among all of the psychological intervention approaches, which ones produce the largest effects on psychological and physiological adaptation and clinical health outcomes in cancer patients. Based upon recent meta-analyses, it appears that CBT-based SMI approaches are particularly effective, but more so in patients with earlier, nonmetastatic disease, in particular breast cancer (Mirosevic et al. 2019).

The “when” question asks at what point in the post-diagnosis cancer continuum should one intervene with SMIs. While there is growing evidence that these interventions can create changes in stress-related biobehavioral processes for periods up to 12 months in patients with early-stage nonmetastatic disease, it remains to be determined whether they are able to modulate these biobehavioral processes in patients with advanced cancers. Similarly, there are two SMI trials that have shown effects on long-term recurrence and survival in early-stage patients receiving intervention in the postsurgical period (Andersen et al. 2008,  Stagl et al. 2015a). Given the established effects of surgery on stress-related biological processes, the peri-surgical period may be an important point to explore in further SMI trials with cancer patients (Eckerling et al. 2021). This could include recruiting patients just after biopsy-confirmed diagnosis, randomizing them to study conditions either prior to neoadjuvant therapy (which precedes surgery) or just prior to surgery (a period of heightened anxiety and stress) to test intervention effects on biobehavioral processes pre-/post-surgery, or recruiting patients after surgery and testing effects pre/post adjuvant therapy to see if early inoculation has lasting effects.

The “where” question concerns the setting for delivery of SMIs and it requires us to consider a variety of implementation issues. As we have noted, extended interventions requiring weekly group attendance over several months may not be practical within the context of primary oncology treatment, and ongoing trials are testing briefer forms and remote delivery platforms (using tablets and broadband connection) to determine if they show comparable effects to their longer and in-person versions. More work should also be conducted testing the effects of embedding SMIs (in-person or remotely delivered) into adjuvant therapy settings such as preparation for radiation treatment (simulation visits) and the chemotherapy infusion suite, where patients are attending multiple treatment sessions and are often isolated from their family and social support system (Biagioli et al. 2017, Mosher et al. 2012).

In terms of the “for whom” question, we need more information on which subgroups of patients are likely to benefit the most from SMIs. There is good evidence that patients with greater cancer-specific distress (Wang et al. 2018), pessimism (Antoni et al. 2001), and other forms of psychosocial adversity (Schneider et al. 2009) show the greatest effects of these interventions on psychological adaptation. However, there is no evidence that these host characteristics can predict SMI effects on physiological adaptation and long-term clinical health outcomes (Antoni & Dhabhar 2019). It is also important to uncover biomedical (e.g., tumor phenotype and immune system status) and sociodemographic factors that identify patients most likely to show psychological, physiological, and health benefits. Elsewhere it has been pointed out that it might also be helpful to test which host factors predict the strongest effects (and in the most cost-effective manner) of one SMI approach over another (e.g.,relaxation versus CBT versus β-adrenergic blockade) (Antoni & Dhabhar 2019). As we learn about host factors that predict optimal SMI effects, we can make use of in-depth psychosocial screening with patient-reported outcomes and biomarker assessments to optimize patient triaging in the spirit of precision oncology care.

## SUMMARY POINTS

The multiple stressors that cancer patients must manage, from the point of diagnosis and treatment decisions, through surgical and adjuvant treatments, and into survivorship, require a substantial amount of both psychological and physiological adaptation.

1. Stress exerts effects on multiple systems, including the sympathetic nervous system and the hypothalamic-pituitary-adrenal (HPA) axis, and it may affect immune processes relevant to the surveillance and progression of cancer (e.g., immune cell adhesion and trafficking, lymphocyte proliferation, macrophage responses, inflammation, angiogenesis, natural kill cell cytotoxicity).
2. Stress management interventions (SMIs) teach skills to help cancer patients and survivors manage and reduce stress and mitigate its deleterious impact on emotional well-being and health.
3. Cognitive behavioral stress management (CBSM) is an SMI that incorporates cognitive behavioral therapy (CBT) skills and relaxation techniques to improve quality of life and reduce symptoms. CBSM integrates core CBT principles and practices, such as cognitive restructuring, behavioral activation, and relaxation techniques like diaphragmatic breathing, progressive muscle relaxation, and meditation/imagery.
4. Randomized controlled trials in cancer survivors have demonstrated that CBT-based SMIs improve quality of life, social support, relaxation, coping skills, and benefit finding and reduce depressive symptoms, anxiety, and emotional distress. These interventions also mitigate stress-related biological changes during treatment,including reduced cortisol; decreased circulating inflammatory markers and reversal of leukocyte nuclear NFκB binding and proinflammatory/prometastatic gene expression; and increased Thelper type 1 cytokine production, lymphocyte proliferation, and leukocyte interferon family gene expression. Finally, CBT-based SMIs have been associated with less depression and better quality of life as well as longer overall and disease-free survival up to 15 years after treatment, which is proportional to intervention-associated reductions in inflammatory signaling during and after initial cancer treatment.
5. With growing evidence that CBT-based SMIs can help cancer patients manage cancerrelated stress and improve psychological and physiological adaptation and health outcomes,future research should focus on (*a*) the role that central nervous system processes play in SMIs, (*b*) the effects of pharmacologic interventions to directly modulate stress biology, (*c*) the effects of SMIs on long-term mental and physical health outcomes, and (*d*) how to extend the reach of SMIs to diverse and underserved populations.

## FUTURE ISSUES

Issues that should be considered in future research include:

1. Increasing the inclusiveness and diversity of cancer populations studied in SMI research and development;
2. Examining the effects of SMIs on most vulnerable groups of patients and survivors (e.g., individuals who are obese, older, have cardiometabolic or other comorbidities);
3. Understanding the role of psychosocial and physiological adaptation processes in advanced cancers and developing novel SMIs to optimize quality of life and health outcomes;
4. Understanding how SMIs affect central nervous system (CNS) processes, neuroendocrine mediators, and changes in peripheral physiology, multiple microbiomes (e.g., gut-brain axis), and carcinogenic processes;
5. Examining how stress processes modulate the cancer–aging bidirectional loop and designing SMIs to slow cancer-accelerated aging from mind to cells;
6. Blending SMIs with health promotion interventions (physical activity, diet/nutrition, medication management) and pharmacologic interventions (β-blockers) in phased care during cancer treatment;
7. Developing and testing evidence-based SMIs that are specifically designed to be integrated before (prehab) and during cancer treatment;
8. Identifying determinants of risk in order to stratify cancer patients and survivors by risk profile to personalize/individualize care (consistent with the precision medicine movement);
9. Leveraging technology (electronic health records, virtual reality, gamification) to identify cancer patients and survivors and effectively deploy SMIs; and
10. Optimizing research–private sector partnerships to implement SMIs into cancer centers as exemplars of sustainable health care.

## Footnotes

1 Readers may consult the National Cancer Institute’s *Dictionary of Cancer Terms* at https://www.cancer.gov/publications/dictionaries/cancer-terms/.

## Abbreviations

QoL: quality of life
SNS: sympathetic nervous system
NE: norepinephrine
β-AR: beta-adrenergic receptor
HPA: hypothalamicpituitary-adrenal
VEGF: vascular endothelial growth factor
IL: interleukin
IFN: interferon
TNF: tumor necrosis factor
CTRA: conserved transcriptional response to adversity
SMI: stress management intervention
CBT: cognitive behavioral therapy
CBSM: cognitive behavioral stress management
CAA: cancer accelerated aging
